# From East to West: A Narrative Review of Healthcare Models in India and the United States

**DOI:** 10.7759/cureus.43456

**Published:** 2023-08-14

**Authors:** Supritha Chintamaneni, Puja Yatham, Sarah Stumbar

**Affiliations:** 1 Department of General Medicine, Jagadguru Sri Shivarathreeshwara Medical College, Mysore, IND; 2 Department of Rehabilitation Medicine, Herbert Wertheim College of Medicine, Miami, USA; 3 Department of Family Medicine, Herbert Wertheim College of Medicine, Miami, USA

**Keywords:** healthcare models, healthcare challenges, us healthcare, indian health system, global healthcare systems

## Abstract

The global healthcare landscape is fraught with quality, cost, equity, and innovation challenges. Despite this, successful healthcare interventions have emerged from unexpected locations. In India, the eradication of certain communicable diseases, the expansion of access to primary care, and the implementation of innovative methods such as telemedicine have demonstrated the potential for community-centered care. In the United States (US), improvements in healthcare quality, accessibility, and the utilization of medical technology, such as the incorporation of telehealth and artificial intelligence, have highlighted opportunities for technological innovation in healthcare delivery. This manuscript reviews the history and development of healthcare systems in India and the US, highlighting each system's strengths, weaknesses, lessons learned, and opportunities for improvement. By examining both systems, we strive to promote a healthcare model that incorporates lessons from each country to improve community-centered care and ultimately provide equitable access to all.

## Introduction and background

The global health system has experienced exponential growth over the last decade due to consistent efforts by diverse transnational actors [1-4] committed to public health, advancing human rights, addressing humanitarian emergencies, and supporting global development. As per a study done in 2018, there are 203 global health organizations. Some of the notable players include The Wolcott Foundation, the Bill & Melinda Gates Foundation, the World Health Organization (WHO), Tuberculosis and Malaria, the Global Health Council, the U.S. Centers for Disease Control and Prevention (CDC), and the United Nations Children’s Fund (UNICEF) [5]. Public-private partnerships account for just over 9% of all identifiable actors, compared to only 0.5% of charitable organizations [3]. The Bill & Melinda Gates Foundation [6] wields considerable influence and collaborates with WHO campaigns on a global scale.

These 203 organizations have headquarters in 73 cities across 16 countries, but it is worth noting the predominant presence of these headquarters in high-income nations, particularly since more than half are based in the United States (US). This raises concerns about an unequal distribution of healthcare actor leadership globally. Among these actors, 61.6% prioritize improving health as their sole primary goal, while 38.4% have additionally stated primary goals. This draws attention to the intricate relationship between health and other international policy issues, such as economic growth and environmental protection. Most global health centers are primarily found in developed countries, even though developing countries suffer the most from preventable diseases [5]. The lack of global health centers in developing countries is further exacerbated by a scarcity of funds, limited other resources, and varied levels of political approval, leading to critical differences in health outcomes nationally and internationally. The double-edged sword for healthcare systems worldwide lies in resource allocation, which is frequently biased toward the private sector and leads to unequal healthcare access. Other factors impacting health outcomes include a lack of healthcare professionals and their migration to developed countries, limited access to care, and socioeconomic issues such as gender inequality and poverty [7,8].

To optimize healthcare systems, these global health organizations are involved in projects related to healthcare financing, enhancing education and training for healthcare professionals, and investing in better technology and new infrastructure. A radical approach instigating poverty reduction and educational equality must be taken to improve health outcomes and reduce the gaps in healthcare access and the effects of social and cultural issues impacting health [8]. For instance, initiatives that provide access to clean water, nutritious food, and secure housing in communities facing social determinants of health can enhance health outcomes [9]. In addition, investing in easily accessible and high-quality education can provide individuals with fundamental health literacy, allowing them to make informed decisions on their health [10].

This narrative review aims to cast light on two prominent healthcare systems, India and the US, emphasizing their strengths, weaknesses, lessons learned, and opportunities for improvement. The healthcare systems of both countries are intricately connected to and influenced by cultural and social contexts. India is confronted with a restricted availability of education and healthcare facilities in rural areas and a significant poverty line. Due to this notable gap in wealth, India has leveraged its experience to create innovative and cost-efficient models [11]. On the contrary, the US has enhanced access to education and healthcare, leading to the integration of advanced medical technology [12]. Therefore, the choice of India and the US is especially pertinent due to the diversity of their healthcare models, the uniqueness of their challenges, and their potential to provide valuable insights into community-centered care and equitable access to healthcare. The comparative analysis presented in this article aims to gather lessons from the Indian and US healthcare systems to inform the development of a more robust and enhanced healthcare system that draws on best practices from across the world.

## Review

Healthcare system in India

Healthcare Successes in India

In the last decade, the healthcare system in India has achieved noteworthy successes, such as eradicating poliomyelitis, yaws, and maternal and neonatal tetanus [13]. India boasts the Universal Immunization Programme (UIP), one of the world's most comprehensive routine childhood vaccination programs. Implemented by the Ministry of Health and Family Welfare in 1978, UIP has significantly decreased the frequency of polio, measles, and tetanus. It significantly advanced India's healthcare system by emphasizing public health and disease prevention [14].

The Indian National Health Mission (established in 2013) significantly raised healthcare awareness nationwide; it includes the National Rural Health Mission (launched in 2005) and the National Urban Health Mission (launched in 2013). The main goal of this mission is to achieve universal access to equitable, affordable, and quality healthcare services that are accountable and responsive to people's needs [15]. Healthcare reforms in India began in 2005 through programs such as the National Rural Health Mission to strengthen rural health services and provide partial financial protection for healthcare to vulnerable families. New proposals made in 2017 promised to pave the way for universal health coverage (UHC). The National Health Policy 2017 laid the groundwork for the latest wave of reforms, which asks for strategic sourcing of secondary and tertiary care from the public and private sectors and comprehensive primary healthcare (PHC) [16].

The COVID-19 epidemic has acted as a catalyst for healthcare reforms in India by highlighting the shortcomings of the existing healthcare system [17,18]. In response to the pandemic, the Indian government realized the need for action and implemented several measures, including higher investment in public health facilities. One such initiative, "Ayushman Bharat" aims to make the country more self-reliant by investing in essential life-saving equipment, such as personal protective equipment (PPE), ventilators, hospital infrastructure, intensive care unit (ICU) beds, oxygen supply, and higher quality laboratory facilities. Telemedicine has also gained popularity in India, allowing for the delivery of healthcare services using information and communication technology. The Indian government has introduced e-sanjeevani, a nationwide teleconsultation service, to help with this transition. Through this service, patients can receive medical advice through audio and video, providing access to healthcare for even those in the country's remotest areas [19].

The Mohalla clinics are a group of state-run community clinics in Delhi that have attracted much national and international attention, including from medical professionals at the state level [11,20]. Mohalla Clinics and similar community clinics across India have made remarkable strides in providing accessible and affordable PHC to underprivileged populations. The medical staff at these clinics have actively addressed social determinants of health (SDOH), such as gender-based violence and alcoholism. These clinics' successes have helped ignite efforts to allocate resources to expanding and improving healthcare services. As a result, India's political and policy discourse on healthcare has changed for the better [21].

The Aravind Eye Care System is another sustainable model developed in Southern India specifically for delivering high-quality, efficient cataract surgery. Founded in 1976, this group of specialty eye hospitals treats more than 3.8 million patients annually, and its facilities undertake over 400,000 ocular procedures each year, two-thirds of which are cataract surgeries. The system's emphasis on sustainability, efficiency, excellent quality, and low cost allows it to offer heavily subsidized treatments for most patients. A study done on the model demonstrates how cataract surgery can be highly cost-effective at just US$195. If this system's model were applied nationwide, 30 million Indians with cataract-related vision loss could get surgery on one eye for under three billion dollars. This would generate an estimated net economic benefit of over $13 billion. Increasing the number of cataract operations from 2,000 to 10,000 per million people yearly would cause a net financial gain of approximately five billion dollars [22]. In conclusion, India's healthcare system has made significant strides over the past several decades, achieving notable successes in eradicating diseases, expanding access to primary care, and implementing telemedicine services.

Healthcare Challenges in India

The Indian healthcare system faces various challenges in delivering quality care to its diverse population. These challenges can be summarized as the five A's: awareness, access, availability, affordability, and accountability [23].

The lack of knowledge about health issues among the population, also known as low health awareness, is a significant public health concern. Several factors contribute to low health awareness, such as poor educational status and inadequate healthcare system focus on education. For instance, studies have shown that only one-third of antenatal mothers had sufficient knowledge about breastfeeding practices [24]. Only 11.3% of adolescent girls in urban Haryana knew about important reproductive health issues [25]. Moreover, a review on geriatric morbidity reported that only 20.3% of participants knew the common causes of prevalent illnesses and their prevention [26]. However, interventions aimed at increasing awareness have yielded positive outcomes. For example, a behavioral change intervention in Bihar and Jharkhand improved awareness and perceptions about abortion [27].

Access to healthcare is defined as the right or opportunity to benefit from healthcare services. However, physical reach, availability, supply, and utilization of healthcare services can limit access, especially in rural areas with limited infrastructure and facilities. Even when healthcare facilities are physically accessible, inadequate infrastructure and personnel can lead to poor quality care [23].

A central challenge is the availability of healthcare personnel across India. This human resource crisis is caused by vacant positions and a preference for working in areas with better infrastructure and facilities. According to a 2011 study, India had approximately 20 healthcare workers per 10,000 people, with allopathic doctors accounting for 31%, nurses and midwives for 30%, pharmacists for 11%, Ayurveda, Yoga, Unani, Siddha, and Homeopathy (AYUSH) practitioners for 9%, and other health workers for 9%. India faces a severe shortage of qualified healthcare professionals, especially in rural areas with few incentives to work [28]. This also perpetuates access barriers.

The issue of healthcare affordability is a significant concern, with the private sector controlling the industry and household spending accounting for approximately 75% of healthcare costs. Therefore, this increase in funding would enable the development of essential infrastructure in underserved areas, improving access to healthcare services, facilities, and personnel [29]. Addressing this challenge will require national and local measures to increase access to affordable healthcare. One critical step is for the government to increase its spending on healthcare from the current level of less than 2% of the gross domestic product (GDP) to around 5% to 6% [30].

Healthcare accountability refers to healthcare institutions' and professionals' responsibility to provide high-quality services and uphold the professional and ethical standards of employers and society [31]. In recent years, the healthcare system has been plagued by multiple tragedies, including those at Gorakhpur Hospital, Chhattisgarh sterilization centers, the Bhubaneswar Hospital fire, the Kolkata Hospital tragedy, and the Erwadi mental asylum tragedy. These disasters represent instances in which healthcare management and infrastructure inadequacies led to substantial loss of life. To discuss these examples individually, the Gorakhpur Hospital incident involved inadequate oxygen supply management; the Chhattisgarh sterilization centers incident revealed negligence during sterilization procedures; the Bhubaneswar Hospital fire revealed insufficient fire safety measures; the Kolkata Hospital tragedy revealed substandard medical practices; and the Erwadi mental asylum tragedy revealed a lack of adequate care and safety measures for mentally ill patients. These occurrences have raised the question of accountability and regulations in disasters. Sadly, physicians are frequently suspended or terminated without a comprehensive investigation into the root cause of the problem [32].

The five A's described above present significant obstacles to realizing public health in India. We must acknowledge and address these challenges to optimize the country's health and combat the detrimental effects of illness on individuals and communities. To tackle the problem of access to high-quality healthcare, it is crucial to identify and analyze potential barriers within the financial, geographic, social, and systemic domains [23]. Some strategies include awareness campaigns to educate patients about preventative health, outreach programs to improve access to healthcare services, and leveraging telehealth technologies.

Despite its recent advances and innovative programs, India's healthcare system continues to experience severe inequalities. While some urban institutions provide excellent medical care and attract healthcare tourists, many others struggle to provide quality and affordable healthcare. India's healthcare system faces persistent health challenges, including low government spending on health, a high incidence of infectious diseases like measles and tuberculosis, a rising burden of non-communicable diseases like diabetes and hypertension, a shortage of healthcare workers, and a lack of emphasis on PHC [13]. Unfortunately, the availability of healthcare services and medications in India is unreliable, leading to significant uncertainty for patients. Poor referral linkage exacerbates this problem by forcing many people to seek treatment at higher-level government health facilities, even for minor illnesses like fever, cough, or upper respiratory infections. This has led to overcrowding, long wait times, inadequate service, and dissatisfaction with public health facilities [11,20].

In conclusion, the healthcare system in India has demonstrated notable advancements in disease eradication, vaccination initiatives, and telemedicine services. However, it continues to face obstacles related to inadequate health awareness, limited accessibility, affordability, and accountability concerns. Therefore, it is crucial to undertake collaborative initiatives aimed at achieving equitable healthcare for all, fostering a healthier nation.

Healthcare system in the USA

Healthcare Successes in the USA

The US healthcare system has experienced many successes in enhancing access to care, patient safety, development of medical technology, application of artificial intelligence, management of chronic diseases, and public awareness. The Patient Protection and Affordable Care Act (ACA), passed by the US Congress in March 2010, establishes a comprehensive and inclusive health insurance system by promoting cooperation between employers, citizens, and the government. Its overarching goal is to develop a comprehensive and inclusive health insurance system. The ACA focuses heavily on primary and preventative care, recognizing their crucial role in promoting overall wellness. The ACA has been successful in improving healthcare quality, increasing accessibility, and reducing unnecessary healthcare costs [33].

Proponents of the ACA emphasize its positive impact on access to healthcare for populations, including those with diabetes and traumatic injuries, due to the Medicaid expansion component [34-36]. The Dependent Care Provision of the ACA has increased insurance coverage in many aspects, including for young adults with cancer [37]. Additionally, tying Medicare payments to readmission rates has incentivized healthcare providers to improve the quality of care to reduce readmissions [38]. Proponents also think the ACA made it easier for public health nurses to focus on preventative healthcare, integrate primary care with broader public health, and coordinate care more efficiently [39]. Overall, the ACA has achieved significant successes in the American healthcare system through healthcare quality and affordability advancements by improving access to care, expanding insurance coverage, promoting preventative health measures, and incentivizing wellness programs.

Another significant advancement in the US healthcare system is the utilization and development of medical technology, particularly in the COVID-19 era. The economic impacts of the pandemic compelled the government to adopt novel technological solutions to enhance operational efficiency [40]. In early 2020, telehealth emerged as an indispensable tool facilitating remote patient diagnosis and treatment. Patients communicated their symptoms through chat boxes or video conferencing to physicians, who provided prompt care. Many healthcare providers now have access to internet-based care tools and remote monitoring, which enable them to stay informed about their patients' illnesses and progress, even if they are homebound [41,42].

Another technological advancement in the US is Artificial Intelligence (AI). According to a recent survey done by the Health Management Academy, 47.5% of healthcare systems currently use AI solutions to address workforce challenges [43]. The US Food and Drug Administration (FDA) has authorized several AI-based products, and hospitals and health systems are increasingly implementing AI-based technology. Medical AI can assist in clinical decision-making, such as prescribing medications or reading radiological images [12]. Based on a retrospective analysis involving 652 patients, the convolution and recurrent neural networks, which are based on AI systems, demonstrated accuracy rates of 92.3% and 82.8%, respectively in identifying malignant and benign pulmonary nodules [44]. These findings indicate that AI holds significant potential and efficacy in the diagnostic process of pulmonary nodules. Moreover, in the future, AI and robotic telemedicine will have the potential to provide clinical care, patient monitoring, and patient diagnostics in off-the-grid locations [45]. During the COVID-19 pandemic, robots cleaned hospital rooms and provided public services like delivering food to quarantined households [46]. As a result, many believe that robots will play a more significant role in restricting human interactions while generating new opportunities in the tech sector and replacing manufacturing jobs. However, it's important to acknowledge that while these advancements show potential, many are still in their early stages and not yet prevalent in the majority of US hospitals. Overall, the US healthcare system has made and is still making significant strides in using technology and innovation to enhance healthcare delivery, improve patient outcomes, and reduce costs, highlighting its potential for future success.

Healthcare Challenges in the USA

Although the US healthcare system has several notable strengths, it has several significant shortcomings, including inadequate access to care, health disparities, high healthcare spending, and unequal distribution of resources and outcomes. The growing importance of medical imaging in diagnosing and treating diseases has brought about significant advancements, but it has also raised concerns regarding healthcare spending and associated challenges. Although medical imaging has been a significant advancement in diagnosing and treating various diseases, it has also presented several challenges, including overdiagnosis, incidental findings, exposure to harmful radiation, and increased healthcare costs. Research estimates indicate that up to 30% of imaging tests may be unnecessary, resulting in a significant loss of $30 billion yearly in the US economy [47]. Moreover, compared to 10 other nations on different measures, the US ranked one or two in the number of computed tomography (CT) and magnetic resonance imaging (MRI) scans conducted per 1000 people [17,48].

Another significant healthcare challenge the US faces today is achieving equity in an evolving healthcare system. Despite efforts to reduce healthcare disparities, unequal frequency and burden of various diseases persist among different racial and ethnic groups. These differences are often present even after accounting for socioeconomic inequalities and healthcare access. According to a study by the Institute of Medicine, clinical contact often reveals evidence of prejudice, uncertainty, and stereotyping contributing to healthcare inequities. It is crucial to raise awareness of these disparities among the public and the healthcare system, promote evidence-based recommendations to facilitate equity in care, and foster a more diverse healthcare workforce to address these issues [49].

The prevalence of mental illness in the US has seen a substantial rise over the last 15 years resulting in a fall in life expectancy from suicide, opioid overdose, and alcoholic liver cirrhosis [50]. Racial-ethnic minority groups in the US also face significant barriers to accessing mental health care, contributing to major disparities in treatment quality and outcomes [51]. These individuals are 20-50% less likely to initiate mental health service use and 40-80% more likely to prematurely discontinue treatment [52]. Although the ACA has tried to mitigate obstacles for certain minority groups, discrepancies still exist [53]. One way to effectively overcome racial prejudice is to use cultural competency in diagnosis and treatment by taking into account the patient's socioeconomic background. For example, the cultural formulation model, as discussed by Jarvis et al. in this publication, assists physicians in accurately diagnosing psychiatric conditions and developing treatment plans that are applicable across many cultural boundaries [54].

While AI is believed to have the potential to revolutionize healthcare, numerous ethical and legal challenges are associated with AI-driven healthcare. Additionally, medical AI may be taught in improper settings, with subpar methods, or insufficient data, leading to errors in diagnosis and treatment. One major challenge with AI is that the current legal framework discourages doctors from acknowledging the potential benefits of AI. There is no case law on liability concerning medical AI, primarily since AI is relatively new to clinical practice [55]. In conclusion, the US healthcare system has advantages, including skilled professionals, but also faces challenges such as access, health inequities, racial disparities, and ethical dilemmas posed by AI, requiring a concerted approach that prioritizes patient outcomes and appropriate use of technology.

## Conclusions

In conclusion, a comparison of the healthcare systems in India and the US shows a complicated terrain of achievements and obstacles. Although both nations have achieved tremendous progress in some areas, their healthcare systems still have issues limiting their ability to function effectively to promote the overall health of the population.

Achievements within the US healthcare system encompass enhanced patient safety, treatment access, and medical technology use, including the incorporation of AI. These developments have helped to improve healthcare quality, broaden accessibility, and cut wasteful spending. However, it is crucial to understand that success in certain areas does not always translate into overall success in other facets of healthcare, as seen with over-expenditure. Despite having few resources, the Indian healthcare system has shown creative methods. Initiatives like community clinics and tested models like the Aravind Eye Care System have shown how critical it is to address socioeconomic determinants of health and provide accessible, reasonably priced healthcare, especially for underserved communities. These methods provide valuable insights that may be applied to healthcare systems globally.

It is essential to take note of the strengths of both the Indian and the US healthcare systems to develop better systems that benefit everyone. To provide fair access and address health inequities, a genuinely strong healthcare system should use technology to spread community initiatives and pharmaceutical advances to the whole population. We may set the goal for healthcare systems to prioritize patient outcomes, innovation, accessibility, and equality by embracing effective elements from both models and encouraging global cooperation. With notable players in the international health scene, such as WHO, CDC, and UNICEF, lending their voices to address healthcare disparities, we can foster a collective effort toward building more equitable and effective healthcare systems worldwide.
